# Circadian Reprogramming by Combined Time-Restricted Feeding and Exercise Improves Metabolic Homeostasis in Diabetes

**DOI:** 10.3390/metabo16040257

**Published:** 2026-04-11

**Authors:** Qingxin Li, Guodong Zhang, Sugao Zhou, Yanli Xie

**Affiliations:** 1 School of Physical Education and Health, Yibin University, Yibin 644000, China; 2 Department of Physical Education, Shanxi Agricultural University, Jinzhong 030801, China; 3 College of Life Sciences, Shanxi Agricultural University, Jinzhong 030801, China

**Keywords:** circadian, time-restricted feeding, exercise, metabolic homeostasis, diabetes

## Abstract

Background: Circadian disruption exacerbates type 2 diabetes mellitus (T2DM). Time-restricted feeding (TRF) and exercise (EX) improve metabolic health, but their combinatory effect remains unclear. This study investigated whether combined TRF and EX additively ameliorates metabolism via circadian reprogramming in db/db mice. Methods: Eight-week-old male db/db mice were assigned to control (Con), diabetic model (DM), TRF (8 h feeding window), EX (treadmill, 60 min/day, 5 days/week), or combined TRF + EX groups for 8 weeks (*n* = 8/group). Body weight, glucose/insulin tolerance, and 24 h energy metabolism (CLAMS) were assessed. Mitochondrial function, oxidative stress, inflammation, and expression of mitophagy (Pink1, Park2, Bnip3, Fundc1) and thermogenic (Ucp1, Pgc1a, Prdm16, Cidea) genes were measured. Results: Compared with the con group, DM mice showed obesity, hyperglycemia and blunted circadian metabolic rhythm. The TRF and EX groups improved these defects. Specifically, combined TRF + EX reduced fasting blood glucose from 25.3 ± 3.1 mmol/L (DM) to 13.2 ± 1.8 mmol/L (*p* < 0.05), body weight from 49.8 ± 2.5 g to 39.5 ± 1.7 g (*p* < 0.05), and body fat percentage from 45.6 ± 3.2% to 32.1 ± 2.2% (*p* < 0.05). GTT area under the curve (AUC) decreased from 3711.0 ± 186.5 (DM) to 2118.0 ± 112.4 (*p* < 0.05), and ITT AUC decreased from 2617.5 ± 135.8 to 1260.0 ± 68.9 (*p* < 0.05). Notably, the combination of TRF + EX produced greater effects than either intervention alone: body weight, fasting blood glucose, and glucose/insulin tolerance were greatly improved (*p* < 0.05). In addition, compared with the DM group, the diurnal metabolic amplitude and phase were improved in the TRF or EX group; the combination group showed further improvements in these parameters. Furthermore, TRF and EX each resulted in significantly higher expression of key thermogenic genes (Ucp1, Pgc1a, Prdm16, Cidea) in white adipose tissue (WAT) and brown adipose tissue (BAT) (*p* < 0.05), and the TRF + EX group showed the highest expression levels. Combined intervention also restored skeletal muscle SOD activity (31.2 ± 2.9 U/mg prot vs. DM 20.1 ± 2.5 U/mg prot, *p* < 0.05) and reduced serum TNF-α (28.5 ± 4.5 pg/mL vs. DM 65.8 ± 8.5 pg/mL, *p* < 0.05) and IL-6 (21.6 ± 3.8 pg/mL vs. DM 50.3 ± 7.1 pg/mL, *p* < 0.05). Conclusions: TRF + EX additively restores metabolic homeostasis in diabetes by re-entraining circadian energy rhythms, improving mitochondrial quality, and activating adipose thermogenesis, supporting further investigation of integrated lifestyle timing as a potential therapeutic strategy.

## 1. Introduction

Type 2 diabetes mellitus (T2DM) is closely linked to lifestyle-induced circadian disruption and metabolic dysregulation, making interventions that target circadian timing particularly relevant [1,2]. Its pathogenesis is intricately linked to lifestyle factors, particularly overnutrition and physical inactivity, which disrupt energy homeostasis and circadian rhythms [3,4]. While pharmacological management remains a cornerstone, limitations such as side effects, cost, and incomplete restoration of metabolic health underscore the critical need for effective non-pharmacological interventions [5,6].

Time-restricted feeding (TRF), a dietary regimen that limits daily food intake to a consistent 8–12 h window without calorie restriction, reinforces endogenous circadian rhythms, improves insulin sensitivity, promotes autophagic flux, and reduces adiposity through mechanisms involving circadian hormone secretion (e.g., insulin, glucagon) and metabolic switching from glucose to lipid oxidation during the fasted state [7,8]. Concurrently, regular physical exercise, particularly when performed at a consistent time of day, reinforces circadian rhythms in peripheral tissues such as skeletal muscle, and is a well-established intervention for improving insulin sensitivity and mitochondrial function [9,10,11]. Both interventions independently target core defects in T2DM, yet their mechanistic pathways are distinct and potentially complementary [7,8,9,10,11].

The db/db mouse, a leptin receptor-deficient model, faithfully replicates key features of human T2DM and metabolic syndrome, including severe obesity, hyperglycemia, insulin resistance, and adipose tissue dysfunction, making it an ideal model for investigating metabolic interventions [12,13]. Previous research has extensively documented the individual benefits of exercise training or various dietary regimens, including caloric restriction, in this model [14,15]. However, studies on the non-calorie-restrictive paradigm of TRF, particularly in combination with exercise, remain limited and only a few studies have explored combined lifestyle interventions in this context [16,17]. A critical gap exists in understanding whether the temporal alignment of feeding (TRF) and activity (exercise) creates additive or synergistic effects that surpass either intervention alone. To our knowledge, this study is among the first to systematically evaluate the combined effects of TRF and exercise on circadian energy metabolism dynamics, mitochondrial quality control, and thermogenic programming in db/db mice. Specifically, the combined impact on circadian energy metabolism dynamics, mitochondrial quality control in insulin-sensitive tissues like skeletal muscle, and the thermogenic programming of white and brown adipose tissue has not been comprehensively explored.

We hypothesize that the combination of TRF and moderate-intensity exercise will produce superior improvements in systemic metabolism in db/db mice compared to single interventions. This additive effect is postulated to arise from coordinated enhancements in circadian entrainment of metabolism, mitochondrial efficiency and turnover via mitophagy, and attenuation of oxidative stress and inflammation. This study aims to systematically evaluate this combinatorial approach, providing mechanistic insights into how integrated lifestyle modifications can be optimized to combat metabolic disease.

## 2. Materials and Methods

### 2.1. Experimental Animals and Grouping

This study was conducted between April and November 2024. Thirty-two 8-week-old male db/db mice and eight age-matched male control m/m mice were obtained from Beijing View Solide Technology Co., Ltd. (Beijing, China). The mice were housed individually in cages under specific pathogen-free conditions, maintained at a constant temperature (23 ± 1 °C) and humidity (30–40%), with a 12 h light/dark cycle (lights on at 7:00 am and off at 7:00 pm). Sample size was determined using G*Power software (version 3.1.9.7) based on a preliminary study, with α = 0.05, power = 0.80, and an estimated effect size of 1.2 for fasting blood glucose (*n* = 8/group). All procedures were conducted in accordance with the National Institutes of Health Guide for the Care and Use of Laboratory Animals. The protocol was approved by the Animal Ethics Committee of Beijing View Solide Technology Co., Ltd. (Approval No.: VS212400909).

**Figure 1:**
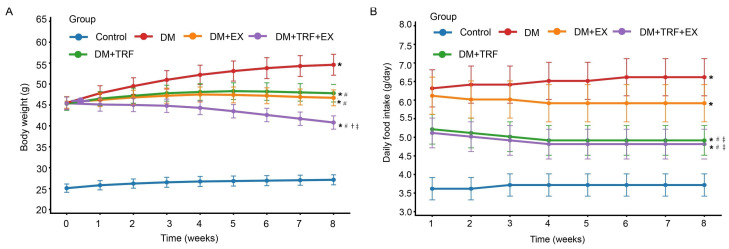
Changes in body weight and food intake of mice in each group. (**A**) Weekly body weight (g) changes in mice in each group. (**B**) Daily and weekly food intake (g) changes in mice in each group, respectively. Data are presented as means ± standard deviation. * *p* < 0.05 vs. Con; # *p* < 0.05 vs. DM; † *p* < 0.05 vs. DM + TRF; ‡ *p* < 0.05 vs. DM + EX (*t* test).

Following a one-week acclimatization period, the mice were divided into groups. The eight m/m mice served as the control group (Con) [16]. The thirty-two db/db mice were randomly allocated into four groups (*n* = 8 per group): the model group (DM), the exercise intervention group (DM + EX), the time-restricted feeding group (DM + TRF), and the combined exercise and time-restricted feeding group (DM + TRF + EX). Randomization was performed using a computer-generated random number sequence. Investigators involved in outcome assessments were blinded to group allocation. All mice in the exercise groups underwent a 5-day treadmill adaptation period (10 m/min, 0% incline, 10 min/day) before the start of the formal intervention. The control wild-type mice had ad libitum access to a standard chow diet (12% kcal from fat, 28% from protein, 60% from carbohydrate; SPF-F02-002). The DM group mice also had ad libitum access to a standard chow diet. The DM + TRF group mice were subjected to time-restricted feeding (food access was limited to an 8 h window, starting 2 h after the onset of the dark phase, daily). Time-restricted feeding with an 8 h feeding window is widely used in mouse studies and has been shown to be safe and humane without causing malnutrition or excessive stress. Mice naturally consume most of their food during the dark phase, and restricting access to 8 h aligns with their innate feeding behavior. Previous studies have demonstrated that TRF does not induce hunger-related stress markers (e.g., corticosterone elevation) when the feeding window is placed during the active phase [18]. Total daily food intake was measured, and no significant differences in total caloric intake were observed between the DM + TRF and DM groups (Figure 1B), confirming that TRF did not involve caloric restriction. The DM + EX group mice performed moderate-intensity treadmill exercise daily at a fixed time (3 h after the onset of the dark phase). A motorized treadmill (DF-PT101, Beijing Jitai Yuancheng Technology Co., Ltd., Beijing, China) was used. The exercise protocol consisted of running on a motorized treadmill for 60 min per day, 5 days per week, at a speed of 15 m/min and a 10% incline (approximately 5.7 degrees, where 10% refers to a 10 cm rise per 100 cm horizontal distance). The exercise intensity (15 m/min at 10% incline) corresponds to approximately 65–70% of VO_2_max based on previous validation in db/db mice [19]. This intensity (65–70% VO_2_max) is classified as moderate-to-high intensity in db/db mice and has been previously shown to be safe and effective for improving metabolic parameters without causing excessive stress or injury [20]. The treadmill speed was gradually increased over the first 20 min: 10 m/min for 10 min, 12 m/min for 10 min, and 15 m/min for the remaining 40 min. During exercise, mice were encouraged to run with gentle prodding (using a soft brush) was used instead of electric shock to minimize stress, as stress can confound metabolic outcomes. This method has been validated in previous studies [21]. The exercise protocol was designed with reference to a previous study [11], with minor adjustments made based on the mice’s performance during the adaptation week. Although conventional recommendations suggest handling during the light (rest) phase to minimize stress, our study aimed to align interventions with the natural circadian rhythm of mice, which are nocturnal. Performing TRF and exercise during the dark (active) phase reinforces endogenous circadian timing and is more translationally relevant to human lifestyle interventions (where feeding and activity occur during wakefulness). To minimize handling stress, all procedures were performed under dim red light and with consistent handling routines [22]. The DM + TRF + EX group mice received both the TRF and EX interventions described above. All mice were maintained under a 12/12 h light/dark cycle for an 8-week intervention period. A schematic diagram of the experimental timeline is provided in Figure S1.

### 2.2. Sample Collection

At the end of the intervention period, mice were euthanized by cervical dislocation. To account for circadian variation, all tissue collections were performed at the same time of day (Zeitgeber time 2–4). Serum, epididymal white adipose tissue (WAT), interscapular brown adipose tissue (BAT), and tibialis anterior (TA) muscle were collected.

### 2.3. Measurement of Body Weight and Food Intake

Body weight was monitored weekly throughout the 8-week experimental period using an electronic balance (Sartorius, Goettingen, Germany). Total weekly food intake was recorded by weighing the remaining chow daily, and the average daily food intake per mouse was calculated as (total weekly intake)/7.

### 2.4. Glucose Tolerance Test (GTT) and Insulin Tolerance Test (ITT)

Prior to testing, mice were transferred to clean cages and fasted for 5 h with free access to water. After fasting, body weight was measured and recorded. The tip of the tail was sterilized and clipped with sterile scissors to collect blood. The first drop of blood was discarded to avoid potential contamination with tissue fluid. The second drop was used for immediate blood glucose measurement with a glucometer (Roche) to establish the baseline (0 min). Mice then received an intraperitoneal injection of either glucose (2 g/kg body weight for GTT) or insulin (0.75 U/kg body weight of recombinant human insulin (Novolin R, Novo Nordisk) for ITT). Blood samples were subsequently collected from the tail vein at 15, 30, 45, 60, and 120 min post-injection. The tail was gently squeezed for each sampling, followed by disinfection and hemostasis. Blood glucose levels at each time point were immediately measured with the glucometer and recorded. The area under the curve (AUC) for glucose during GTT and ITT was calculated using the trapezoidal method.

### 2.5. Measurement of Energy Metabolism in Mice

Energy metabolism was analyzed using metabolic cages. The Comprehensive Lab Animal Monitoring System (CLAMS; PhenoMaster, TSE Systems) was used to continuously monitor parameters including hourly oxygen consumption (VO_2_), total oxygen consumption, hourly carbon dioxide production (VCO_2_), total carbon dioxide production, respiratory exchange rate (RER), and hourly energy expenditure. Mice were individually housed in metabolic cages for 48 h, with data from the second 24 h cycle used for analysis to allow for acclimatization. Mice maintained their respective dietary patterns during the monitoring period. The acquired metabolic data were subsequently analyzed. Circadian rhythm parameters (amplitude and phase) were analyzed using cosine curve fitting (circacompare package in R) to provide quantitative comparisons.

### 2.6. Enzyme-Linked Immunosorbent Assay (ELISA)

At the end of the experiment, blood was collected via the orbital sinus and centrifuged at 3000 rpm for 10 min to obtain serum. Commercial ELISA kits were used to determine the levels of superoxide dismutase (SOD), adenosine triphosphate (ATP), and mitochondrial respiratory chain complex I activity in skeletal muscle homogenates, as well as serum levels of tumor necrosis factor-alpha (TNF-α) and interleukin-6 (IL-6). The testing kit companies are Nanjing Jiancheng Bioengineering Institute, and the following kits were used: SOD (A001-2-2), ATP (A095-1-1), Complex I (A089-1-1), TNF-α (H052-1-2), IL-6 (H007-1-1). All assays were performed in duplicate strictly according to the manufacturers’ instructions.

### 2.7. Reverse Transcription-PCR (RT-PCR)

Muscle and adipose tissues were lysed using Trizol reagent (AG21102, Precision Biotechnology, New Taipei City, Taiwan). Total RNA was extracted from the samples using the RNAex Pro Reagent (Accurate Biology, Changsha, China). RNA purity and concentration were assessed using a NanoDrop 2000 (Thermo Fisher, Waltham, MA, USA), with A260/A280 ratios between 1.8 and 2.0. First-strand cDNA was synthesized using the Evo M-MLV Reverse Transcription Kit (Accurate Biology, Changsha, China). RT-qPCR was performed in triplicate using the SYBR Green PCR Kit (Accurate Biology, Changsha, China) on a real-time PCR system (Bio-RAD, Hercules, CA, USA). GAPDH was used as the internal reference gene. The relative mRNA expression levels of *Pink1*, *Park2*, *Bnip3*, *Fundc1*, *Ucp1*, *Pgc1a*, *Prdm16*, and *Cidea* were calculated using the 2^−ΔΔCt^ method. Primer sequences are listed in Table 1.

### 2.8. Statistical Analysis

Data were analyzed using R software (version 4.5.1) with packages “ggplot2”, “car”, and “emmeans”. Normality was assessed using the Shapiro–Wilk test, and homogeneity of variances was assessed using Levene’s test. All data met normality assumptions (*p* > 0.05) except where noted, and appropriate non-parametric tests were used for those variables. Data were expressed as mean ± standard deviation. For comparisons among multiple groups, one-way ANOVA followed by Tukey’s HSD post hoc test when assumptions were met was used. For comparisons between two groups, an unpaired two-tailed *t*-test was applied. For repeated measures data (GTT, ITT, and metabolic cage data), a two-way repeated measures ANOVA with group and time as factors was used, followed by Bonferroni-corrected post hoc tests. A *p*-value < 0.05 was considered statistically significant.

## 3. Results

### 3.1. Body Weight and Food Intake

In this study, 8-week-old mice were subjected to interventions including a standard diet, time-restricted feeding (TRF), and moderate-intensity treadmill exercise (EX). The testing concluded after 8 weeks. Compared to the control (Con) group, the DM group showed a significant increase in body weight (*p* < 0.05), demonstrating the typical obese phenotype of db/db mice. Relative to the DM group, body weight gain was comparably and effectively suppressed in both the DM + TRF and DM + EX groups, with body weight at 8 weeks being significantly lower than that of the DM group (*p* < 0.05). Starting from 3 week, the body weight control effect in the DM + TRF + EX group was significantly greater than that in either single-intervention group (DM + TRF or DM + EX), and this difference gradually widened over the subsequent weeks. By 8 week, the body weight in the DM + TRF + EX group approached baseline levels and was significantly lower than that in both the DM + TRF and DM + EX groups (Figure 1A). Compared to the DM group, the average daily food intake per week was significantly reduced in both the DM + TRF and DM + TRF + EX groups (*p* < 0.05) (Figure 1B).

### 3.2. Effects of Different Interventions on Body Fat and Glucose Metabolism in Diabetes

**Figure 2:**
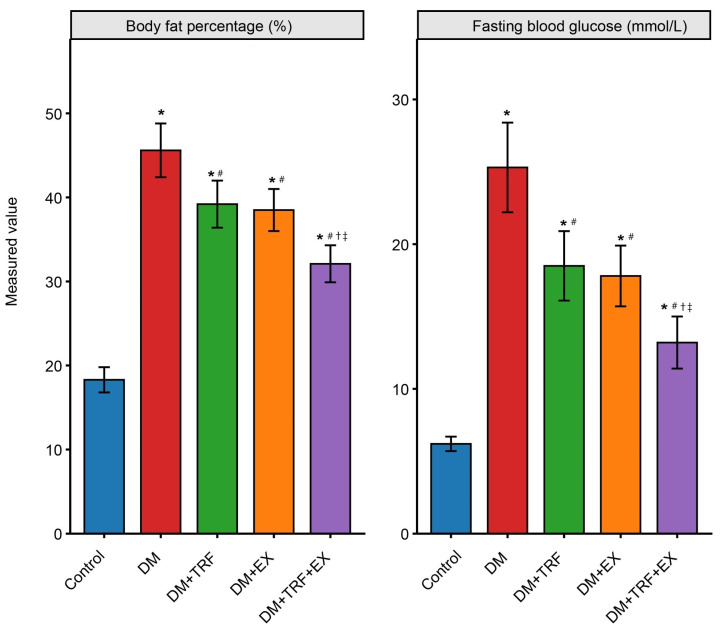
Body fat percentage and fasting blood glucose levels in each group of mice. Data are presented as means ± standard deviation. * *p* < 0.05 vs. Con; # *p* < 0.05 vs. DM; † *p* < 0.05 vs. DM + TRF; ‡ *p* < 0.05 vs. DM + EX (one-way ANOVA with Tukey’s post hoc test).

All three intervention strategies produced varying degrees of improvement in the metabolic parameters of DM mice. Following time-restricted feeding (TRF) intervention (DM + TRF group), the mice exhibited a significant reduction in both body weight (45.1 ± 2.1 g vs. DM 49.8 ± 2.5 g) and body fat percentage (39.2 ± 2.8% vs. DM 45.6 ± 3.2%) compared to the DM group (*p* < 0.05), along with a significant improvement in fasting blood glucose (18.5 ± 2.4 mmol/L vs. DM 25.3 ± 3.1 mmol/L, *p* < 0.05). Similarly, exercise intervention (DM + EX group) also significantly decreased body weight (43.8 ± 1.9 g), body fat percentage (38.5 ± 2.5%), and fasting blood glucose (17.8 ± 2.1 mmol/L) compared to DM (*p* < 0.05). Notably, the combined TRF and exercise intervention (DM + TRF + EX group) demonstrated the most potent metabolic benefits. Mice in the combined intervention group showed significantly lower body weight (39.5 ± 1.7 g), body fat percentage (32.1 ± 2.2%), and fasting blood glucose (13.2 ± 1.8 mmol/L) not only compared to the DM group (*p* < 0.05) but also relative to either single intervention group (*p* < 0.05) (Figure 2). In particular, the fasting blood glucose level in the combined intervention group was reduced compared to the DM group, indicating an additive effect of the two interventions.

### 3.3. GTT and ITT

**Figure 3:**
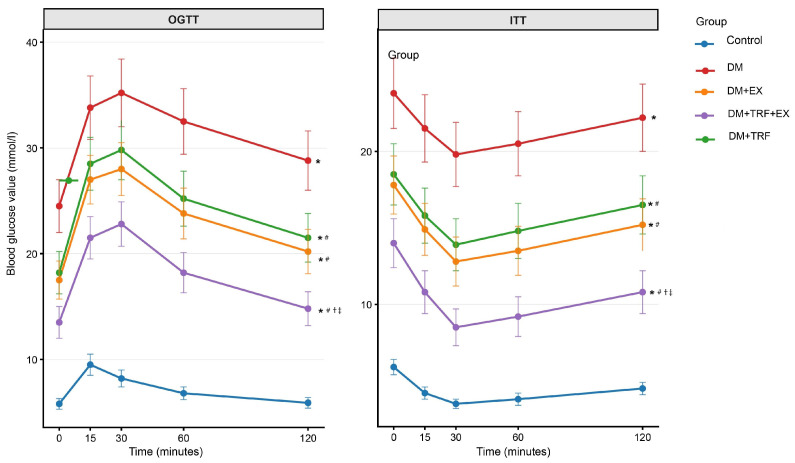
Results of the OGTT and ITT. Data are presented as means ± standard deviation. * *p* < 0.05 vs. Con; # *p* < 0.05 vs. DM; † *p* < 0.05 vs. DM + TRF; ‡ *p* < 0.05 vs. DM + EX (two-way repeated measures ANOVA with Bonferroni post hoc tests).

In the GTT, compared to the control group, blood glucose levels in the DM group were significantly elevated at all time points (*p* < 0.05), indicating severely impaired glucose tolerance. Both single interventions (TRF or exercise alone) significantly ameliorated this glucose intolerance in DM mice (*p* < 0.05). Notably, the combined TRF and exercise intervention demonstrated the most pronounced improvement, with blood glucose values at all time points being significantly lower than those in the DM group (*p* < 0.05) and, at multiple time points, superior to either single intervention group (*p* < 0.05). In the ITT, DM mice exhibited significant insulin resistance, as evidenced by a significantly slower rate of blood glucose decline compared to the control group (*p* < 0.05). While both single interventions significantly improved insulin sensitivity (*p* < 0.05), the combined intervention resulted in the greatest restoration of insulin sensitivity, showing the greatest reduction in blood glucose at each time point and performing significantly better than either single intervention (*p* < 0.05) (Figure 3). The area under the curve (AUC) for glucose during GTT and ITT was calculated using the trapezoidal method. The DM + TRF + EX group showed significantly lower AUC compared to DM (*p* < 0.05) and significantly lower than either single intervention group (*p* < 0.05) (see Tables S1 and S2).

### 3.4. Combined Intervention Effectively Ameliorates Oxidative Stress and Inflammatory Levels

**Figure 4:**
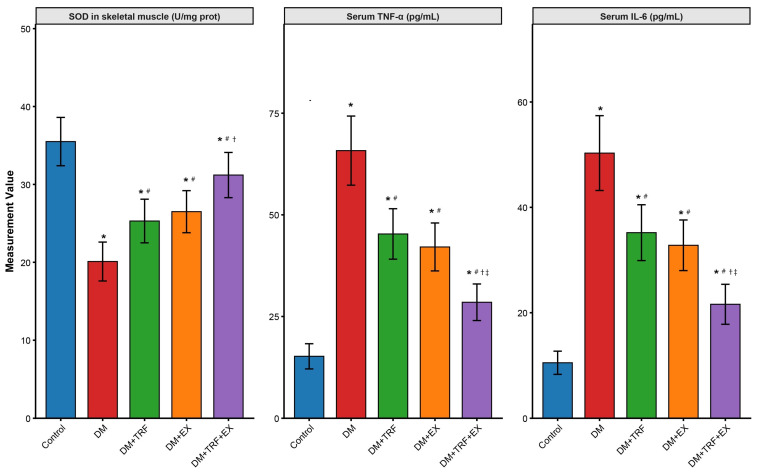
Changes in oxidative stress and inflammatory cytokine levels in each group of mice. Data are presented as means ± standard deviation. * *p* < 0.05 vs. Con; # *p* < 0.05 vs. DM; † *p* < 0.05 vs. DM + TRF; ‡ *p* < 0.05 vs. DM + EX (one-way ANOVA with Tukey’s post hoc test).

To explore the potential mechanisms underlying the metabolic improvements, we further examined markers of oxidative stress and inflammation. As shown in Figure 4, compared with the control group, DM mice exhibited a significant reduction in the activity of the antioxidant enzyme superoxide dismutase (SOD) in skeletal muscle (20.1 ± 2.5 U/mg prot vs. Con 35.5 ± 3.1 U/mg prot, *p* < 0.05), alongside significantly elevated serum levels of TNF-α (65.8 ± 8.5 pg/mL vs. Con 15.2 ± 3.1 pg/mL, *p* < 0.05) and IL-6 (50.3 ± 7.1 pg/mL vs. Con 10.5 ± 2.2 pg/mL, *p* < 0.05). Either TRF intervention (DM + TRF group) or exercise intervention (DM + EX group) partially reversed these abnormalities, significantly increasing SOD activity (25.3 ± 2.8 U/mg prot and 26.5 ± 2.7 U/mg prot, respectively, *p* < 0.05 vs. DM) and decreasing TNF-α (45.3 ± 6.2 pg/mL and 42.1 ± 5.9 pg/mL, *p* < 0.05 vs. DM) and IL-6 (35.2 ± 5.3 pg/mL and 32.8 ± 4.8 pg/mL, *p* < 0.05 vs. DM). However, the combined TRF and exercise intervention (DM + TRF + EX group) demonstrated an additive effect, resulting in the most substantial restoration of SOD activity (31.2 ± 2.9 U/mg prot) and the greatest reduction in serum TNF-α (28.5 ± 4.5 pg/mL) and IL-6 (21.6 ± 3.8 pg/mL). These metrics were significantly better than those in either single intervention group (*p* < 0.05) (Figure 4). This indicates that the combined intervention possesses an additive advantage in mitigating oxidative stress and suppressing chronic low-grade inflammation.

### 3.5. Impact of Interventions on Dynamic Energy Metabolism Characteristics

**Figure 5:**
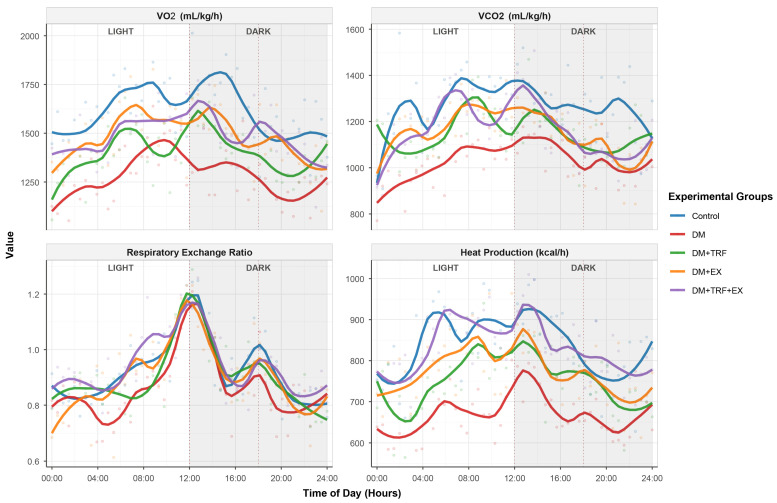
24 h metabolic cage monitoring in diabetic and intervention groups. gray shading: dark phase (12:00–24:00), dotted lines: feeding times.

To further elucidate the effects of the interventions on dynamic energy metabolism characteristics, we assessed real-time energy metabolism parameters in mice using a 24 h metabolic cage monitoring system. As shown in Figure 5, mice in the diabetic (DM) group exhibited significant metabolic rhythm disturbances and an overall decline in energy metabolism. Their oxygen consumption (VO_2_), carbon dioxide production (VCO_2_), RER, and heat production were significantly lower than those in the control group at various time points throughout the day and night (*p* < 0.05) (Supplementary Figure S1). Furthermore, the amplitude of their metabolic curve was significantly reduced, and a phase delay was observed, indicating impaired metabolic flexibility and circadian rhythmicity. Both single interventions, TRF or EX alone, partially improved the metabolic parameters in DM mice, restoring their average levels and rhythmicity to some degree. Notably, the combined TRF and EX intervention produced the most pronounced additive effect: mice in the combined intervention group (DM + TRF + EX) showed the greatest restoration in the 24 h mean values of VO_2_, VCO_2_, heat production, and RER. Their metabolic curve most closely resembled that of the control group, particularly demonstrating higher metabolic peaks during the dark/active phase and postprandial periods. Quantitative rhythm analysis using cosine curve fitting confirmed that the combined intervention restored circadian amplitude and phase to a greater extent than either single intervention (Figure S2). These data suggest that the combined intervention most effectively reshapes the circadian rhythm of whole-body energy metabolism and enhances metabolic flexibility, which may be one of the key mechanisms underlying its improvement in glucose and lipid metabolism.

### 3.6. Combined Intervention Significantly Improves Skeletal Muscle Mitochondrial Function and Energy Metabolism

**Figure 6:**
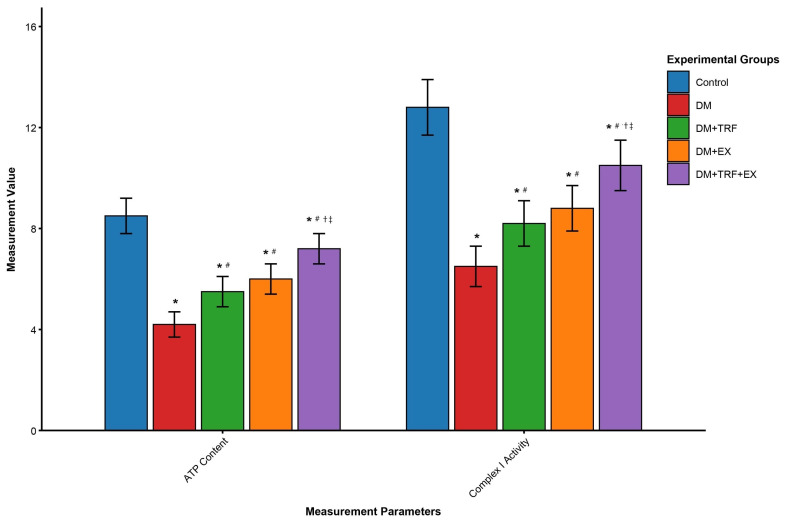
Effects of different interventions on skeletal muscle energy metabolism. Data are presented as means ± standard deviation. * *p* < 0.05 vs. Con; # *p* < 0.05 vs. DM; † *p* < 0.05 vs. DM + TRF; ‡ *p* < 0.05 vs. DM + EX (one-way ANOVA with Tukey’s post hoc test).

To further investigate the cellular energetic basis of the observed systemic metabolic improvements, we evaluated skeletal muscle mitochondrial function. As shown in Figure 6, compared to the control group, DM mice exhibited significant reductions in skeletal muscle ATP content (4.2 ± 0.5 nmol/mg prot vs. Con 8.5 ± 0.7 nmol/mg prot, *p* < 0.05) and mitochondrial respiratory chain complex I activity (6.5 ± 0.8 U/mg prot vs. Con 12.8 ± 1.1 U/mg prot, *p* < 0.05), indicating severely impaired mitochondrial oxidative phosphorylation. Both single interventions, TRF or EX alone, partially reversed this deficit, significantly elevating ATP content (5.5 ± 0.6 nmol/mg prot and 6.0 ± 0.6 nmol/mg prot, respectively, *p* < 0.05 vs. DM) and complex I activity (8.2 ± 0.9 U/mg prot and 8.8 ± 0.9 U/mg prot, *p* < 0.05 vs. DM). However, the combined TRF and EX intervention (DM + TRF + EX group) demonstrated the strongest additive effect, with ATP content restored to 7.2 ± 0.6 nmol/mg prot and complex I activity to 10.5 ± 1.0 U/mg prot, both significantly higher than either single intervention group (*p* < 0.05). This indicates that the combined intervention most effectively enhances skeletal muscle mitochondrial energy production efficiency, providing a crucial cellular energetic foundation for the observed systemic metabolic improvements.

### 3.7. Combined Intervention Effectively Activates Skeletal Muscle Mitophagy Pathways

**Figure 7:**
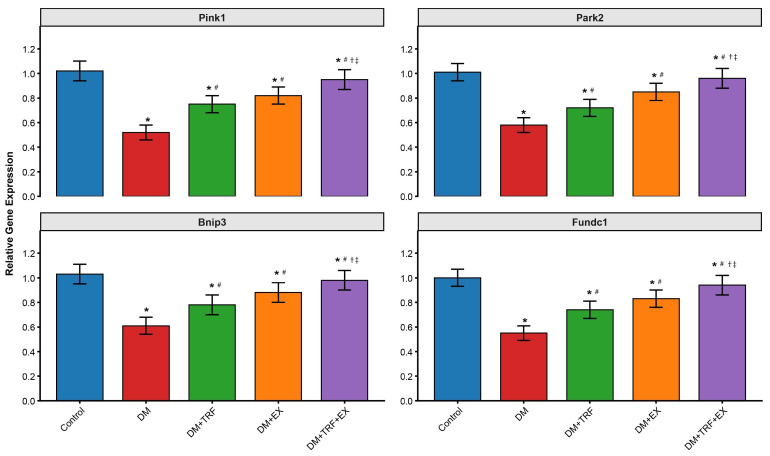
Expression of mitophagy-related genes in skeletal muscle. Data are presented as means ± standard deviation. * *p* < 0.05 vs. Con; # *p* < 0.05 vs. DM; † *p* < 0.05 vs. DM + TRF; ‡ *p* < 0.05 vs. DM + EX (one-way ANOVA with Tukey’s post hoc test).

To elucidate the potential quality control mechanisms underlying the improvement in mitochondrial function, we examined the expression of key regulatory genes involved in mitophagy in skeletal muscle. As shown in Figure 7, compared to the control group, the expression levels of mitophagy-related genes (*Pink1*, *Park2*, *Bnip3*, *Fundc1*) were significantly downregulated in DM mice (*p* < 0.05), suggesting impaired mitochondrial quality control. Both TRF intervention and exercise intervention effectively reversed this trend, significantly upregulating the expression of these genes (*p* < 0.05), with exercise intervention (DM + EX group) showing a slightly stronger activating effect than TRF intervention alone. However, the combined TRF and exercise intervention (DM + TRF + EX group) demonstrated the strongest additive activating effect. The restoration of expression levels for each gene was most pronounced in this group and was significantly higher than that in either single intervention group (*p* < 0.05). These results indicate that the combined intervention most effectively enhances mitophagic activity in skeletal muscle, which may represent a key molecular mechanism by which it promotes the clearance of damaged mitochondria, thereby improving overall mitochondrial function and cellular energy metabolism.

### 3.8. Changes in Thermogenic Gene Expression in WAT and BAT

**Figure 8:**
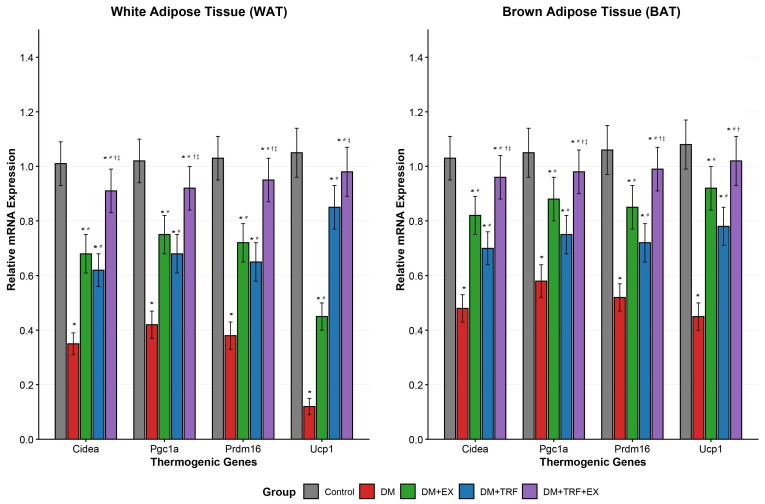
mRNA expression levels of thermogenic genes in white adipose and brown adipose tissues. Data are presented as means ± standard deviation. * *p* < 0.05 vs. Con; # *p* < 0.05 vs. DM; † *p* < 0.05 vs. DM + TRF; ‡ *p* < 0.05 vs. DM + EX (one-way ANOVA with Tukey’s post hoc test).

As shown in Figure 8, the DM model led to significant suppression of key thermogenic genes (*Ucp1*, *Pgc1a*, *Prdm16*, *Cidea*) in both WAT and BAT compared to the control group (*p* < 0.05). This suppression was more pronounced in WAT, with *Ucp1* expression reduced to merely 11.4% of the control level. Individual interventions, either TRF or EX, partially reversed this suppression (*p* < 0.05), yet their effects differed. In WAT, TRF showed a stronger upregulating effect on *Ucp1*, while exercise induced more significant upregulation of genes like *Pgc1a*. In BAT, exercise exhibited a slightly additive effect over TRF in elevating most genes. Remarkably, the combined TRF and EX intervention (DM + TRF + EX group) demonstrated the strongest additive effect across all genes. The expression levels in this group were not only significantly higher than those in the DM group (*p* < 0.05) but also, for most indicators, significantly higher than those in either single intervention group (*p* < 0.05). The gene expression in BAT of the combined group was nearly fully restored to control levels. These findings indicate that the combined intervention most effectively activates the thermogenic program in adipose tissues, which may provide a crucial molecular basis for the observed increase in whole-body energy expenditure.

## 4. Discussion

In this study, we demonstrated that combining time-restricted feeding with moderate-intensity exercise produces additive improvements in systemic metabolism in db/db mice, including enhanced glycemic control, restored circadian energy metabolism, improved mitochondrial function, activated mitophagy, and upregulated adipose tissue thermogenesis. These findings suggest that integrated lifestyle interventions targeting both the timing of energy intake and expenditure may offer a promising non-pharmacological approach for managing metabolic dysfunction.

The superior control of body weight and adiposity in the DM + TRF + EX group aligns with the concept that TRF and EX target energy balance through complementary mechanisms [23]. TRF primarily regulates the timing of energy intake, enhancing circadian hormone secretion (e.g., insulin, glucagon) and promoting metabolic switching from glucose to lipid oxidation during the fasted state [24]. Exercise, conversely, increases total daily energy expenditure and improves skeletal muscle metabolic capacity [24]. Their combination likely creates a pronounced negative energy balance while preserving lean mass, a finding supported by human studies showing greater fat loss with combined diet and exercise [25]. The dramatic 48% reduction in fasting glucose and the robust improvements in GTT/ITT curves suggest a powerful restoration of insulin sensitivity. This may involve enhanced skeletal muscle GLUT4 translocation [26], improved hepatic insulin signaling due to TRF-induced reductions in hepatic steatosis [27], and ameliorated adipose tissue inflammation, which is a key driver of systemic insulin resistance [28].

A novel finding of this study is the profound restoration of circadian metabolic rhythms by the combined intervention. The flattening of VO_2_, VCO_2_, and RER rhythms in DM mice reflects metabolic inflexibility—a hallmark of diabetes [29]. TRF is known to act as a powerful zeitgeber (time cue) for peripheral clocks, particularly in the liver and intestine [30]. Exercise also influences circadian rhythms in peripheral tissues like skeletal muscle [31]. Our data suggest their combination optimally resynchronizes whole-body metabolism, leading to higher amplitude rhythms and appropriate metabolic responses to feeding (higher postprandial peaks). This restored rhythmicity may be fundamental to improving glucose homeostasis and lipid metabolism.

At the cellular level, the combined intervention most effectively reversed skeletal muscle mitochondrial dysfunction. The recovery of ATP content and Complex I activity indicates improved oxidative phosphorylation efficiency. Crucially, this was coupled with the strongest upregulation of mitophagy genes (*Pink1*, *Park2*, *Bnip3*, *Fundc1*). Mitophagy is essential for removing damaged mitochondria, and its impairment contributes to oxidative stress and insulin resistance in T2DM [32]. TRF has been shown to activate autophagy/mitophagy via AMPK and circadian pathways [33]), while exercise induces mitophagy through mechanisms involving AMPK and BNIP3 [34]. The observed additive effects may therefore reflect more efficient mitochondrial turnover, which aligns with the significant reductions in oxidative stress (increased SOD) and inflammation (decreased TNF-α, IL-6). This cleaner mitochondrial pool and less inflamed environment are conducive to improved insulin signal transduction.

Furthermore, the combined regimen powerfully activated the thermogenic program in both white and brown adipose tissue. The upregulation of *Ucp1*, *Pgc1a*, *Prdm16*, and *Cidea*, especially the near-complete restoration in BAT, indicates a shift toward energy-dissipating adipocytes. This “browning” of WAT and activation of BAT can significantly contribute to whole-body energy expenditure [35]. Exercise is a known inducer of FNDC5/irisin, which can stimulate browning [36], while TRF may promote adipose remodeling through β-hydroxybutyrate signaling and sympathetic nervous system activation [37]. Their combined effect suggests a potent stimulation of adaptive thermogenesis, providing a potential molecular basis for the elevated energy expenditure observed in metabolic cages.

This study has several strengths, including the use of a well-established diabetic mouse model (db/db), a randomized and blinded experimental design, and comprehensive metabolic phenotyping using CLAMS to assess circadian energy metabolism. The inclusion of multiple tissue analyses (skeletal muscle, WAT, BAT) and the assessment of both functional (mitochondrial respiration) and molecular (gene expression) endpoints provide a multi-dimensional view of the intervention effects. Additionally, the use of TRF without caloric restriction allows for the specific evaluation of feeding timing as an independent variable, distinct from caloric restriction.

This study has several limitations. First, using only male mice precludes understanding potential sex-specific responses. Second, while we measured key markers, more detailed assessments of tissue-specific insulin signaling (e.g., p-AKT/AKT), mitochondrial dynamics (fusion/fission proteins), and sympathetic nervous system activity would deepen mechanistic insight. Additionally, our conclusions regarding circadian reprogramming, mitophagy activation, and thermogenic remodeling are primarily based on mRNA expression and indirect physiological measurements; further studies using protein-level analyses and tissue-specific knockouts are needed to establish causality. Third, the translational relevance to humans requires confirmation in clinical trials. Future research should investigate the specific molecular crosstalk (e.g., AMPK/SIRT1/PGC-1α pathways, clock gene expression) mediating the additive effects and explore the optimal timing of exercise within the TRF window for maximal benefit.

## 5. Conclusions

In conclusion, our findings demonstrate that the integration of TRF and exercise creates additive metabolic improvements in a murine model of T2DM. These effects are associated with circadian re-entrainment, enhanced mitochondrial quality control via mitophagy, and activation of adipose tissue thermogenesis. This preclinical evidence supports further investigation of combined lifestyle interventions as a potential non-pharmacological strategy for managing metabolic syndrome and T2DM, highlighting the importance of targeting both the timing of energy intake and expenditure.

### Recommendations and Practical Applications

The present findings suggest that combining time-restricted feeding with consistent daily exercise may offer an effective non-pharmacological strategy for improving metabolic health. From a translational perspective, these results highlight the potential value of integrating temporal patterns of both eating and physical activity into lifestyle interventions for individuals with or at risk for type 2 diabetes. Future clinical studies should examine the feasibility, optimal timing, and long-term sustainability of such combined approaches in human populations.

## Figures and Tables

**Table 1:** Primer sequences of gene.

Tissue	Gene Name	Forward Primer	Reverse Primer
skeletal muscle	*Pink1*	TTCTTCCGCCAGTCGGTAG	CTGCTTCTCCTCGATCAGCC
	*Park2*	TCTTCCAGTGTAACCACCGTC	GGCAGGGAGTAGCCAAGTT
	*Bnip3*	TCCTGGGTAGAACTGCACTTC	GCTGGGCATCCAACAGTATTT
	*Fundc1*	CCCCCTCCCCAAGACTATGAA	CCACCCATTACAATCTGAGTAGC
WAT, BAT	Ucp1	AGGCTTCCAGTACCATTAGGT	CTGAGTGAGGCAAAGCTGATTT
	Pgc1a	CGCAGCCCTATTCATTGT	GCATCCTTTGGGGTCTTT
	Prdm16	CCAAGGCAAGGGCGAAGAA	AGTCTGGTGGGATTGGAATGT
	Cidea	TGACATTCATGGGATTGCAGAC	GGCCAGTTGTGATGACTAAGAC
	GAPDH	TGGCCTTCCGTGTTCCTAC	GAGTTGCTGTTGAAGTCGCA

## Data Availability

The original contributions presented in this study are included in this article. Further inquiries can be directed to the corresponding author.

## Supplementary Materials

The following supporting information can be downloaded at: https://www.mdpi.com/article/10.3390/metabo16040257/s1, Figure S1: Experimental flowchart of this study; Figure S2: 24 h metabolic cage monitoring in diabetic and intervention groups; Table S1: AUC values of oral glucose tolerance test (GTT) (mmol/L × min); Table S2: AUC values of insulin tolerance test (ITT) (mmol/L × min).
