# Supplementary material for: Circadian Reprogramming by Combined Time-Restricted Feeding and Exercise Improves Metabolic Homeostasis in Diabetes
Source: Metabolites. 2026 Apr 11;16(4):257. doi: 10.3390/metabo16040257 (PMC13117964; doi:10.3390/metabo16040257)
Supplement: Supplementary file 1 [file metabolites-16-00257-s001.zip › metabolites-4229537-supplementary.pdf]

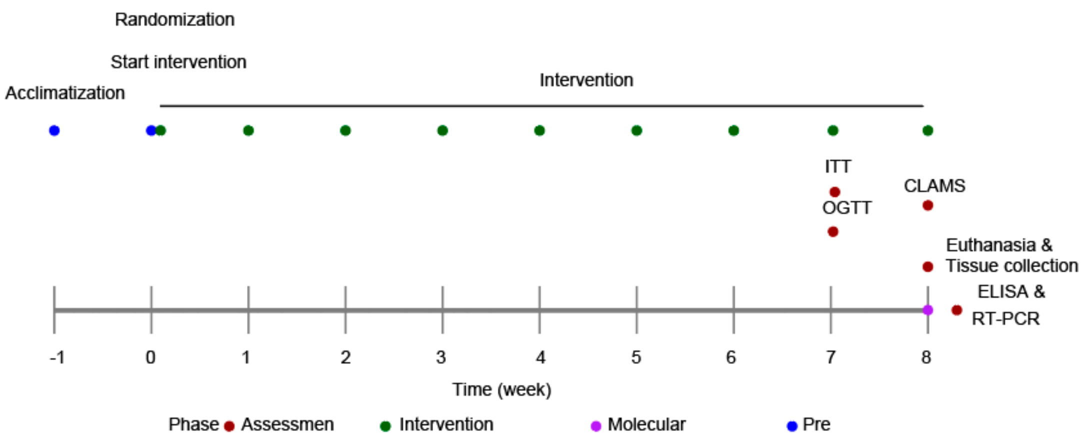

Figure S1. Experimental flowchart of this study

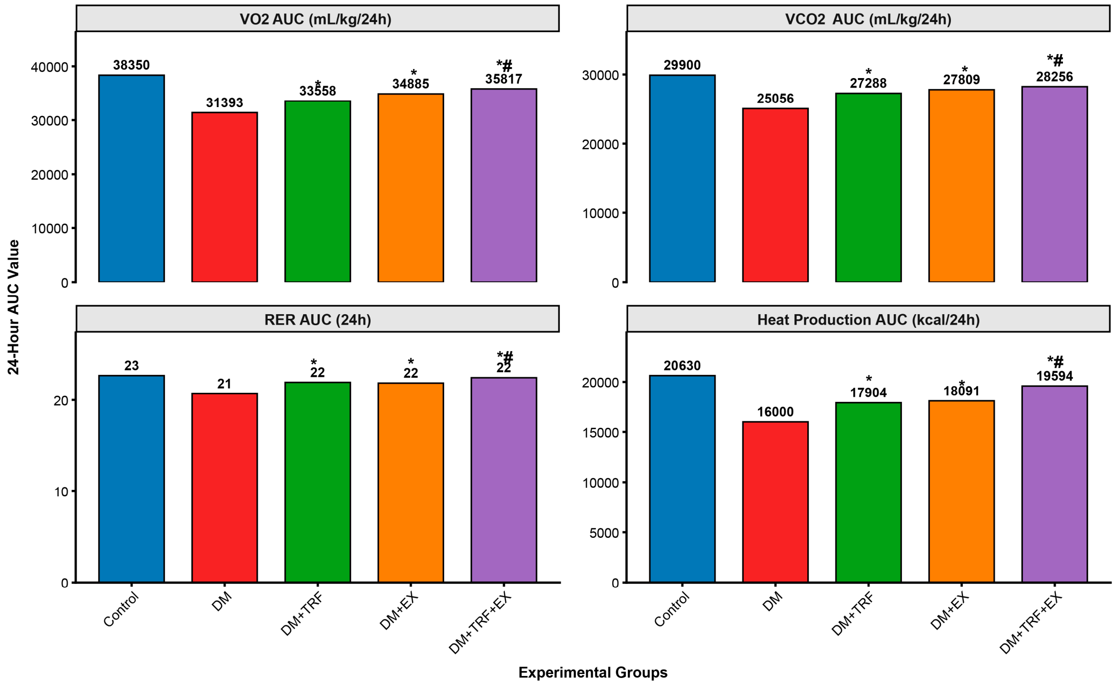

Figure S2. 24-hour metabolic cage monitoring in diabetic and intervention groups. * indicates significant difference compared to DM group ($p < 0.05$), # indicates significant difference compared to DM+EX group ($p < 0.05$).

Table S1. AUC values of oral glucose tolerance test (GTT) (mmol/L × min)

Group	control group	DM group	DM+TRF group	DM+EX group	DM+TRF+EX group
AUC	880.5 ± 45.2	3711.0 ± 186.5	2980.5 ± 152.3*	2838.0 ± 148.6*	2118.0 ± 112.4*#

Data are presented as mean ± standard deviation (n = 8),* indicates significant difference compared to DM group ($p < 0.05$), # indicates significant difference compared to DM+EX group ($p < 0.05$).

Table S2. AUC values of insulin tolerance test (ITT) (mmol/L × min)

Group	control group	DM group	DM+TRF group	DM+EX group	DM+TRF+EX group
AUC	525.0 ± 28.6	2617.5 ± 135.8	1957.5 ± 102.5*	1797.0 ± 94.2*	1260.0 ± 68.9*#

Data are presented as mean ± standard deviation (n = 8),* indicates significant difference compared to DM group (p < 0.05), # indicates significant difference compared to DM+EX group (p < 0.05).
